# Management of Idiopathic Infantile Chylous Ascites

**DOI:** 10.7759/cureus.55965

**Published:** 2024-03-11

**Authors:** Henry Zou, James Van Beynen, Joshua Ritzema

**Affiliations:** 1 Pediatrics, Michigan State University College of Human Medicine, Grand Rapids, USA; 2 Pediatrics, Helen DeVos Children’s Hospital, Grand Rapids, USA

**Keywords:** congenital lymphatic malformation, total parenteral nutrition, drainage, paracentesis, chylous ascites

## Abstract

Chylous ascites is the accumulation of triglyceride-rich lymphatic fluid in the peritoneal cavity. We present the case of a four-month-old male admitted for abdominal distension. A large volume of ascites was confirmed by imaging. Paracentesis indicated chylous aspirate and drainage was performed using a pigtail catheter. Total parenteral nutrition was initiated and fluconazole prophylaxis was implemented for seven days. Twenty-six days after admission, abdominopelvic magnetic resonance imaging showed trace ascites but no signs of lymphatic malformation. He began transitioning to nasogastric feeds with plans to eventually resume oral feeds. This case not only highlights the limitations in our abilities to definitively identify the etiology of pediatric chylous ascites but also demonstrates how chylous ascites management can carefully combine conservative and surgical strategies to optimize patient outcomes.

## Introduction

Chylous ascites (CA) is the accumulation of thoracic and intestinal lymph in the abdominal cavity that manifests as milky, triglyceride-rich peritoneal fluid [1]. It is caused by a disruption to the lymphatic system through lymph flow obstruction or lymph exudation through dilated retroperitoneal vessels, with congenital lymphatic malformations and trauma being the most common etiologies in children [1]. Though a 20-year study published in 1982 found a CA incidence rate of 1/20,000 among admissions at Massachusetts General Hospital (MGH), large-scale epidemiological studies have yet to be conducted since then [2]. This same study found that of the 28 patients admitted for CA at MGH over a 20-year period, only four were children; however, there have been no epidemiological studies on CA incidence in the pediatric population as of this writing [2]. CA is considered a medical emergency as it can reduce the bioavailability of nutrients and immunoglobulins, inducing dehydration, malnutrition, immunosuppression, electrolyte disturbance, and protein-losing enteropathy [1]. Magnetic resonance (MR) lymphangiography and paracentesis are the gold-standard diagnostic methods, but ultrasound (US), computed tomography (CT), and magnetic resonance imaging (MRI) are also commonly used to guide diagnosis [1,3]. Treatment strategies include nutrition management, paracentesis, fluid replacement, infection control, surgical shunts, chyle leak closure, and liver transplantation [4]. Here, we present a case of idiopathic infantile CA.

## Case presentation

A four-month-old male infant (gestational age 40 weeks and 5 days, birth weight 3.86 kg) with a history of bilateral hydroceles presented to the emergency department for abdominal bloating. The abdominal US showed a large volume of ascites, and a kidney, ureter, and bladder X-ray showed bowel displacement compatible with ascites. Furthermore, a chest X-ray showed low lung volumes with mild central atelectasis, and a liver US showed anterograde flow in the portal vein and decreased pulsatility in the hepatic veins. However, venous thromboembolism was ruled out using iliac and inferior vena cava (IVC) US, and the findings of an echocardiogram were within normal limits.

**Figure 1:**
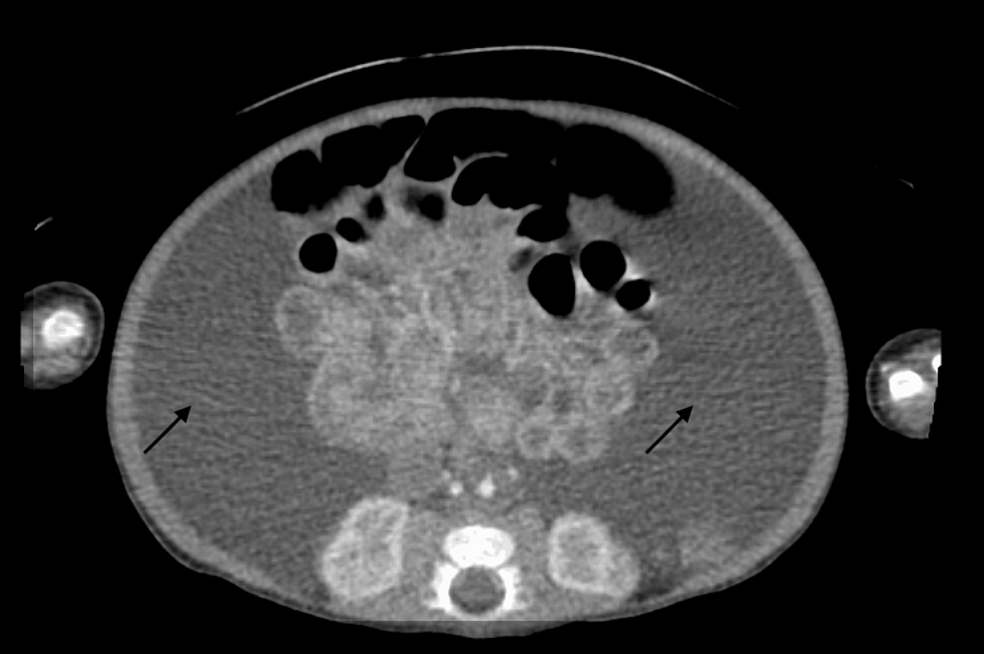
Large volume of chylous ascites on abdominopelvic computed tomography scan. Arrows indicate areas with chylous ascites.

Paracentesis was performed which showed triglyceride levels >4,000 mg/dL consistent with a chylous aspirate, and thoraco-abdominopelvic CT showed a large volume of abdominopelvic ascites that extended through the bilateral inguinal canal into the bilateral scrotum (Figure 1).

Four days after admission, a central line was placed and continuous drainage was performed using a pigtail peritoneal catheter. The patient tested negative for Epstein-Barr virus and cytomegalovirus on antibody tests, ova and parasites on stool tests, and bacterial infection on peritoneal fluid cultures. Nonetheless, intravenous (IV) fluconazole prophylaxis 6 mg/kg every 24 hours (q24h) was initiated for fungal infection risk from the pigtail catheter, which was discontinued after seven days following acceptable trending of immunoglobulin G levels >40 g/L.

One day after central line placement, a “nothing by mouth” diet with total parenteral nutrition and intralipid therapy (TPN/IL) was initiated. Fluid balance was also closely monitored due to high chylous output and was initially replaced using normal saline and fresh frozen plasma according to the Children’s Hospital of Philadelphia Lymphatic Disorders Clinical Pathway guidelines. However, the fluid replacement was discontinued two days later due to decreased chylous output. An Enfaport™ oral diet was trialed but discontinued secondary to increased chylous output, while octreotide was trialed but discontinued due to concerns for potential cell line suppression.

**Figure 2:**
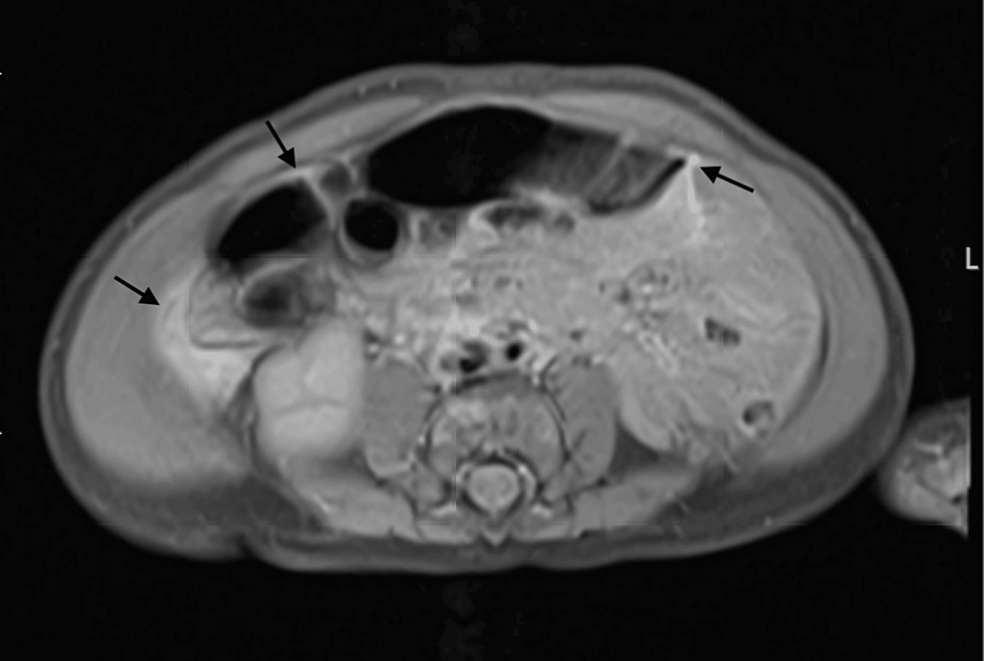
Trace chylous ascites on abdominopelvic magnetic resonance imaging (axial view). Arrows indicate areas with chylous ascites.

A repeat abdominal US showed trace ascites 20 days after initial hospital admission. Meanwhile, iron deficiency anemia was noted using complete blood count and iron studies, so the patient was given three doses of IV ferric gluconate 2 mg/kg q24h and monitored through iron studies which remained unremarkable. Six days later, an abdominopelvic MRI was performed, which also showed trace ascites but no evidence of lymphatic malformation (Figure 2). However, prominent retroperitoneal lymphatic vasculature was noted in the upper abdomen.

His abdominal circumference remained stable on repeat US, and 29 days after initial admission, he began gradually transitioning from TPN/IL to enteral nasogastric feeds with plans to resume oral feeds ultimately.

## Discussion

Pediatric CA has been most commonly associated with congenital lymphatic malformations involving aplasia, hypoplasia, obstruction, or severance of the thoracic duct [5]. Trauma due to parturition, surgery, or abuse is another common cause, and CA has manifested as postoperative complications following congenital heart disease and diaphragmatic hernia repairs [5]. Less common causes include lymphangioma, lymphadenopathy, tumors, infection, and intestinal malrotation [5-7]. Meanwhile, chronic liver disease, cirrhosis, and heart failure can also mediate various types of ascites in pediatric patients [4]. Thus far, investigations into genes involved in lymphangiogenesis have not identified associations with congenital thoracic duct malformations, and over 50% of CA cases in children remain idiopathic [5,6]. Before diagnostic paracentesis in our patient, hepatic, cardiac, and vascular etiologies were ruled out through unremarkable liver, iliac, and IVC US and echocardiogram findings. Trauma was also ruled out based on history. Furthermore, infectious etiologies were ruled out through viral antibody tests, ova and parasite stool tests, and peritoneal fluid cultures. Although abdominopelvic MRI findings showed no evidence of lymphatic malformations, our institution was not equipped to perform MR lymphangiography; consequently, congenital lymphatic malformations in our patient cannot be definitively ruled out.

Treatment of CA focuses on relieving fluid pressure on the diaphragm, reducing the production and accumulation of chyle, and maintaining hydration, adequate nutrition, and electrolyte balance [4,8]. Conservative methods include observation with supportive care, enteral therapy involving high-protein, low-fat diets with medium-chain triglyceride supplementation, TPN, fluid replacement therapy, and somatostatins or octreotide [4,5]. Should conservative treatments fail, surgical intervention including therapeutic paracentesis, drainage, pleuroperitoneal or peritoneovenous shunts, pleurodesis, thoracic duct embolization/ligation, and ligation/surgical closure of chyle leak sites may be indicated [4,5]. A recent case of congenital CA in a preterm female infant born at 33 weeks gestation was successfully treated with paracentesis, octreotide, IV immunoglobulin, nutrition management using Monogen and breast milk, and antibiotics for sepsis [8]. Meanwhile, another case of congenital CA in a five-month-old full-term female infant failed conservative treatment and subsequently underwent surgical removal of adhesions obstructing the thoracic duct [9]. Unfortunately, she died of peritoneal cavity infection, electrolyte imbalance, and malnutrition three months after hospital discharge [9]. Our patient was treated with drainage, TPN/IL, fluid replacement, and enteral therapy. Enfaport-based nutrition support and octreotide were trialed but ultimately discontinued due to CA exacerbation and cell line suppression concerns.

## Conclusions

We present a case of idiopathic CA in a four-month-old male infant successfully managed with drainage, TPN/IL, fluid replacement, and enteral therapy. This case highlights persistent limitations in our abilities to definitively identify the underlying etiology in many cases of pediatric CA and the need for further research on this subject to improve clinical care. Moreover, this case highlights how the management of pediatric CA can carefully combine surgical and conservative strategies to optimize patient outcomes and avoid potentially severe adverse effects.
